# The Sex and Sex Determination in *Pyropia haitanensis* (Bangiales, Rhodophyta)

**DOI:** 10.1371/journal.pone.0073414

**Published:** 2013-08-26

**Authors:** Yuan Zhang, Xing-hong Yan, Yusho Aruga

**Affiliations:** 1 College of Fisheries and Life Science, Shanghai Ocean University, Shanghai, China; 2 Key Laboratory of Exploration and Utilization of Aquatic Genetic Resources, Shanghai Ocean University, Ministry of Education, Shanghai, China; 3 Nishikamata, Tokyo, Japan; Donald Danforth Plant Science Center, United States of America

## Abstract

*Pyropia haitanensis* has a biphasic life cycle with macroscopic gametophytic blade (n) and microscopic filamentous conchocelis (2n) phase. Its gametophytic blades have long been believed to be mainly dioecious. However, when crossing the red mutant (R, ♀) with the wild type (W, ♂), the parental colors were segregated in F_1_ blades, of which 96.1% were linearly sectored with 2–4 color sectors. When color sectors were excised from the color-sectored blades and cultured singly, 99.7% of the color sectors appeared to be unisexual with an equal sex ratio. Although the sex of color sector did not genetically link with its color, the boundaries of both sex and color sectors coincided precisely. About 87.9% of the examined color-sectored blades were monoecious and the percentage increased with the number of color sectors of a blade. The gametophytic blades from each conchocelis strain produced by parthenogenesis of the excised color sectors were unisexual and unicolor, showing the same sex and color as their original sectors. These results indicate that most of the sexually reproduced *Py. haitanensis* blades are monoecious, and their sex is controlled by segregation of a pair of alleles during meiosis of conchospore, forming a sex-sectored tetrad. During the subsequent development of blades, one or two lower cell(s) of the tetrad contribute mainly to rhizoid formation, and rarely show their sexual phenotype, leading to reduced frequency of full sex phenotype of the meiotic blades. Moreover, the aberrant segregations of sex genes or color genes in a few of F_1_ blades were probably due to gene conversions, but there was no sex transfer in *Py. haitanensis*.

## Introduction

*Pyropia haitanensis* (T. J. Chang et B. F. Zheng) N. Kikuchi et M. Miyata [1] is endemic to China and has been commercially cultivated for more than fifty years. Currently, its output accounts for about 75% of the total production of *Pyropia* in China [2]. *Py. haitanensis* has a biphasic life cycle that alternates between macroscopic gametophytic blades (n) and microscopic filamentous conchocelis (2n) phase [3]. It typically undergoes sexual reproduction [4] but does not produce archeospores for asexual reproduction [5]. It has long been believed that most of the gametophytic blades of this species are dioecious and only a few are monoecious according to the visual observations of natural populations [6], [7]. However, in recent years, studies using karyological observation, genetic hybridization and molecular markers indicated that its meiosis takes place during the first two divisions of conchospores, and the initial four cells of the conchosporeling are arranged linearly, forming a meiotic tetrad, which develops into a genotype-sectored blade through subsequent mitosis [8]–[10]. The occurrence position of meiosis for *Py. haitanensis* is the same as that for *Py. yezoensis* [11]–[13] and *Porphyra purpurea* [14]. In addition, the results of cross experiment between a red mutant and the wild type in *Py. haitanensis* showed that the meiotic segregation of parental colors and the ontogenesis of color-sectored blades are very similar to those of *Py. yezoensis* and *P. purpurea* [8] except that the blades of *Py. yezoensis* and *Py. haitanensis* were derived from a linear tetrad [8], [15] while those of *P. purpurea* were derived from a nonlinear tetrad [14]. The sex segregation of *P. purpurea* occurs during meiosis of conchospore, forming monoecious blades [14]. According to these results, it seems likely that the gametophytic blades of *Py. haitanensis* produced by sexual reproduction would be monoecious rather than dioecious [7]. Meanwhile, our previous studies also found that both male and female blades which were regenerated from enzymatically isolated single vegetative cells of the wild-type blades could produce homozygous conchocelis through parthenogenesis if no heterosexual blades were present, and the conchospores released from these homozygous conchocelis would develop into unisexual blades which were fertile and showing uniform color and morphological characteristics, and the blades produced by male-parthenogenesis were all male while those produced by female-parthenogenesis were all female [7]. In addition, karyological observation of the homozygous conchocelis produced by parthenogenesis and their next blade generation demonstrated that the conchocelis had ten chromosomes (2n) and the blades had 5 chromosomes (n), confirming that the conchocelis is derived from agamospore [16] after chromosome's natural double and the meiosis also occurs in germinating conchospores released from the conchocelis [17], [18].

Therefore, the sex of *Py. haitanensis* blades has to be re-examined. In the present study, the segregation of two genetic markers (i.e. color and sex) was studied in F_1_ gametophytic blades produced in the cross between a red mutant (R, ♀) and the wild type (W, ♂) of *Py. haitanensis*. These data were used to better understand sex determination in this species.

## Materials and Methods

### Character trait of parents

In the present study, a wild-type strain (*PT*–*WT*, W, ♂) was used as male parent. Its free-living conchocelis was obtained by the male-parthenogenesis [7] of a male gametophytic blade which was regenerated from a vegetative cell isolated enzymatically from a wild-type blade [19]. All blades of this strain were male, thick, and characterized by red brownish green in color, a strong elasticity, and the presence of numerous marginal denticles. A wild-type gametophytic blade was treated with irradiation of ^60^Co-γ ray, and its vegetative cells were subsequently isolated enzymatically to regenerate into blades [19]. Among the regenerated blades, a female red mutant (*SPY*–*1*, R, ♀) was picked out [19] and its free-living conchocelis (strain) was obtained by its female-parthenogenesis [7], and was used as female parent in the present experiment. All blades of this strain were female and characterized by red in color, thinner blades, high growth rate, weak elasticity, and lack of marginal denticles. The free-living conchocelis of both strains were maintained in the laboratory as described by Yan et al. [19].

### Cross and culture

A male blade and a female blade were respectively selected from offsprings of the parental strains and co-cultured in a flask until zygotosporangia [16] appeared. Zygotospores released from zygotosporangia of the fertilized female blade were collected and grown individually to conchocelis colonies in test tubes at 23±1°C under 10 µmol photons m^−2^ s^−1^ (14L∶10D). When the diameter of the conchocelis colonies reached about 1 cm, they were fragmented in a homogenizer, and inoculated into clean clam shells and cultured for growing shell-boring conchocelis (conchocelis shells). The inoculated shells were then incubated with the MES medium [20] at 23±1°C under 10 µmol photons m^−2^ s^−1^ (12L∶12D). After culture for 10 days, the shells were washed to clear superfluous conchocelis filaments on the shell surface, the culture medium was renewed, and the photon flux density was increased to 20 µmol photons m^−2^ s^−1^. A few weeks later, the culture temperature was increased to 25±1°C without change of other culture conditions. After another two weeks, the culture temperature was increased to 28±1°C, while the photon flux density was decreased to 10 µmol photons m^−2^ s^−1^ (10L∶14D) to induce formation of conchosporangia. Once conchosporangia formed, the conchocelis shells were transferred into a 250 mL Erlenmeyer flask containing 100 mL culture medium, and cultured with aeration in an incubator at 25±1°C under 40 µmol photons m^−2^ s^−1^ (10L∶14D). After 3–5 days of culture, conchospores released from the heterozygous conchocelis were collected, passed gently through a 50 µm nylon mesh filter, and cultured in Petri dishes containing the MES culture medium at 23±1°C under 40 µmol photons m^−2^ s^−1^ (10L∶14D) to obtain F_1_ gametophytic blades. The culture medium was refreshed every week.

### Culture of F_1_ gametophytic blades and color sectors

When F_1_ gametophytic blades reached 2–4 mm long after cultured for about 3 weeks in Petri dishes, all blades were carefully collected using a single-side razor blade. From among them, 1400 blades were randomly selected and inspected under a microscope (Olympus BH, Tokyo, Japan), and the color type and the number of sectors in each blade were recorded. The remaining blades were cultured in several flasks (2000 mL) with aeration at 25°C under 40 µmol photons m^−2^ s^−1^ (10L∶14D).

After 2–3 weeks of further culture, the color-sectored blades grew to be 3–5 cm long and their color sectors (0.5–2 cm long) could be clearly distinguished by naked eyes. Then, 30 color-sectored blades (20 blades with two color-sectors, 6 blades with three color-sectors and 4 blades with four color-sectors) were picked out and cultured singly in different flasks (500 mL) with aeration until each sector of the blade matured to confirm the sex and sexual boundary of the color sectors. In addition, before blades matured, 173 color-sectored blades with macroscopic color sectors (43 blades with two color sectors, 22 blades with three color sectors and 108 blades with four color sectors) were picked out and all of color sectors were excised from them and cultured singly in different flasks (500 mL) with aeration at 25±1°C under 40 µmol photons m^−2^ s^−1^ (10L∶14D) until the spermatangium or female gamete [16] formed. The sex of each color sector was ascertained under a microscope (Olympus CK, Tokyo, Japan) according to such characteristics as shape, size and cell-division formulas of spermatangium and female gamete. When the sex of each color sector was ascertained, the sex and color phenotype of each color-sectored blade were recorded for genetic analysis.

### Sex observation of the parthenogenetic progeny of different sectors from color-sectored F_1_ blades

To test the stability of sex heredity of color sectors, 50 color sectors (10 R (♀), 10 R (♂), 5 R′ (♀), 5 R′ (♂), 5 W (♀), 5 W (♂), 5 W′ (♀) and 5 W ′(♂)) were randomly excised from the immature color-sectored blades 3–5 cm long and were singly cultured. After 20–80 days of further culture, these color sectors underwent parthenogenesis and formed homozygous free-living conchocelis. After 3–4 months of further culture, the free-living conchocelis of each sector were respectively inoculated into clam shells to obtain shell-boring conchocelis as described above. The conchospores released from the mature shell-boring conchocelis were cultured into blade populations, and the sex and color of each population were examined when they matured as described above.

### Statistical analyses

A chi-square (χ^2^) test with Yates correction was applied to analyze the sex and color ratios in the F_1_ gametophytic blades at the 5% significance level. A student's *t*-test was applied to analyze the sex difference among 2-sectored, 3-sectored and 4-sectored blades. *P* value of<0.01 was considered significant.

## Results

### Segregation of parental colors in F_1_ gametophytic blades

**Figure 1:**
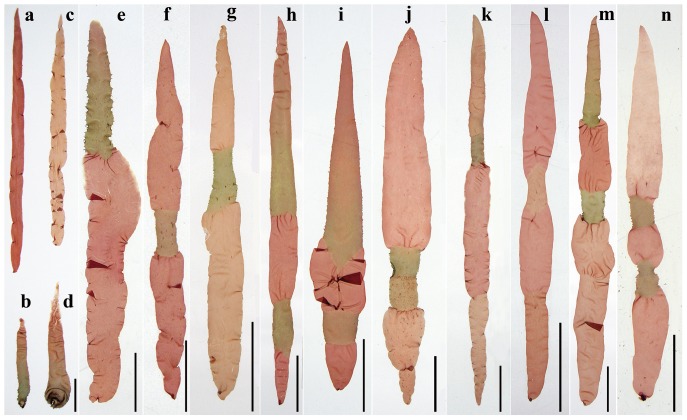
Macrophotographs of F_1_ gametophytic blades developed from conchospores released from heterozygous conchocelis in the cross between a red mutant (R,♀) and the wild type (W,♂) of *Pyropia haitanensis*. *a*–*d*. Single-colored blades: *a*. A red (R) blade; *b*. A wild-type (W) blade; *c*. A near- red (R′) blade; *d*. A near-wild-type (W′) blade; *e*–*l*. Color-sectored blades with 2–4 sectors: *e*. R+W; *f*. R+W′+R; *g*. R′+W+R′; *h*. R+W+R+W; *i*. R+W′+R+W′; *j*. R′+W′+W+R; *k*. R′+R+W+W′; *l*. R′+R+R′+R (without paternal color); *m*-*n*. Color-sectored blade with 5 sectors: *m*. R′+R+W+R+W; *n*. R+W′+R+W′+R′. The color phenotypes are shown from the base to the tip of a blade. *a*–*d* are the same magnification. Scale bars: 1 cm in *a–i* and *k–m*, and 0.5 cm in *j* and *n*.

Four kinds of color sectors, two parental color sectors (W sector and R sector) and two recombinant color sectors (R′ sector and W′ sector) appeared in the F_1_ gametophytic blades developed from the conchospores of heterozygous conchocelis in the present experiment (Fig. 1). R′ sector had the same characteristics as R sector except for the color which was lighter in the former than in the latter. W′ sector was different from W sector only with respect to the color which was more reddish in the former than in the latter. When the F_1_ blades grew to 2–4 mm long, it was confirmed by microscopic observation that 96.1% (1345/1400) of them were color-sectored blades with 2–4 linearly arranged color sectors, and the rest were single-colored blades. The percentage of color-sectored blades with 2, 3 and 4 sectors was 54.4, 33.8 and 7.9%, respectively. Meanwhile, it was also found that each of the color sectors in a blade did not develop evenly. Generally, one or two basal color sector(s) stopped growth or grew very slowly, while one or two uppermost color sector(s) developed into the dominant part of a blade having a blade sector nearly with entire margin. As a result, when the blades grew to 3–5 cm long, the percentage of color-sectored blades decreased to 48.4% (42.2% for 2-sectored blades, and 6.2% for 3- and 4-sectored blades) while that of single-colored blades increased to 51.6%, as observed by naked eyes. However, when observed microscopically, it became clear that about 89.0% of the single-colored blades and 58.0% of the 2-sectored blades had one or two very small color sector(s) in the basal part.

In addition, about 0.8% of the F_1_ blades showed unusual color segregation. A few blades had five color sectors, such as R′+R+W′+R+W (Fig. 1m) and R+W′+R+W′+R′ (Fig. 1n). Some of the 4-sectored blades had only maternal (R) and/or near-maternal (R′) color sectors, such as R′+R+R′+R (Fig. 1l), lacking paternal (W) and/or near-paternal (W′) color sectors, while some of other 4-sectored blades lost one paternal or near-paternal color sector but had more than two maternal or near-maternal color sectors, such as R′+R+W+R (Table 1).

**Table 1:** Phenotypes of color and sex in the color-sectored F_1_ blades produced in the cross between a red mutant (R, ♀) and the wild type (W, ♂) of *Pyropia haitanensis*.

Color phenotype*	Sex type	Blade number	Color phenotype	Sex type	Blade number	Color phenotype	Sex type	Blade number
R+W	♀+♂	11	R+W+R+W	♀+♀+♂+♂	5	R+W′+W+ R′	♀+♀+♂+♂	1
R+W	♂+♀	16	R+W+R+W	♂+♀+♀+♂	2	R+W′+W+ R′	♂+♂+♀+♀	1
R+W	♀+♀	4	R+W+R+W	♀+♂+♂+♀	4	R+W′+R′+W′	♀+♀+♂+♂	1
R+W	♂+♂	9	R+W+R+W	♂+♂+♀+♀	3	R+W′+R +W′	♀+♀+♂+♂	2
R+W′	♂+♀	1	R+W+R+W	♂+♀+♂+♀	2	R+W′+R +W′	♂+♀+♂+♀	3
R+R′	♀+♂	1	R+W+R+W	♀+♀+♀+♀	1	R+W′+R′+W	♂+♀+♀+♂	1
R′+W′	♂+♀	1	R+W+R+W′	♂+♀+♀+♂	4	R+W′+R′+W	♂+♂+♀+♀	1
**Total**		**43**	R+W+R+W′	♂+♀+♂+♀	2	R+W′+R′+W	♀+♀+♀+♀	1
R+W+R	♀+♀+♂	4	R+W+R+W′	♀+♀+♂+♂	1	R+W′+R′+W	♂+♂+♂+♂	1
R+W+R	♀+♂+♀	2	R+W+R+W′	♂+♂+♀+♀	1	R+ R′+R+ R′	♀+♀+♀+♀	1
R+W+R	♂+♀+♀	1	R+W+R+W′	♀+♂+♀+♂	2	R+ R′+R+ R′	♂+♂+♀+♀	1
R+W+R	♂+♂+♀	1	R+W+R′+W′	♀+♀+♂+♂	1	R+ R′+R′+R	♀+♂+♀+♂	1
R+W+R′	♀+♀+♀	1	R+W+R′+W′	♀+♂+♂+♀	1	R′+W+R+W′	♂+♀+♀+♂	2
R+W+R′	♀+♂+♀	1	R+W+R′+W′	♂+♀+♀+♂	1	R′+W+R+W′	♀+♂+♀+♂	1
R+W+R′	♂+♀+♂	1	R+W+R′+W′	♂+♀+♂+♀	2	R′+W+R+W′	♀+♂+♀+♂	3
R+W+R′	♂+♂+♀	1	R+W+R′+W′	♂+♂+♀+♀	1	R′+W+R′+W	♂+♀+♀+♂	1
R+W+W′	♀+♂+♀	1	R+W+R′+W′	♀+♂+♀+♂	2	R′+W+R′+W′	♀+♀+♂+♂	1
(R) +W+W′	(♂+♀)+♀+♂	1	R+W+W′+R	♂+♂+♀+♀	1	R′+W+W′+R	♀+♂+♀+♂	1
R+W+W′	♀+♂+♂	1	R+W+W′+R	♀+♀+♂+♂	1	R′+W+W′+R	♀+♀+♂+♂	1
R+W′+W	♀+♀+♂	1	R+W+W′+R	♂+♂+♂+♂	1	R′+W+ R+W′	♀+♂+♂+♀	1
R′+R+W′	♀+♂+♀	1	R+W+W′+R′	♀+♀+♀+♀	1	R′+W′+W+R′	♀+♂+♀+♂	1
R′+R+W	♂+♂+♂	1	R+W+W′+R′	♀+♂+♀+♂	2	R′+W′+W+R′	♂+♀+♀+♂	1
R′+W+W′	♀+♂+♂	1	R+W′+W+R	♂+♀+♀+♂	2	R′+W′+R+W	♀+♂+♂+♀	2
R′+W′+R	♀+♂+♂	1	R+W′+W+R	♂+♀+♂+♀	1	R′+W′+R+W	♂+♀+♂+♀	2
R′+W′+R	♂+♀+♂	1	R+W′+W+R	♂+♀+♂+♀	1	R′+W′+W+R	♂+♀+♂+♀	2
R′+W′+R′	♂+♂+♀	1	R+W′+W+R	♀+♀+♂+♂	2	R′+W′+W+R	♀+♀+♂+♂	1
**Total**		**22**	R+W′+W+R	♀+♂+♀+♂	1	R′+W′+W+R	♀+♂+♀+♂	2
R′+R+W+R	♀+♀+♂+♂	1	R+W′+W+R′	♀+♂+♀+♀	1	R′+W′+R′+W′	♂+♂+♀+♀	1
R′+R+R′+R	♀+♂+♀+♂	1	R+W′+W+R′	♀+♂+♂+♀	3	R′+W′+R′+W′	♀+♂+♀+♂	1
R′+R+R′+R	♂+♀+♂+♀	1	R+W′+W+R′	♀+♂+♀+♂	1	W+R+W+R	♂+♀+♂+♀	1
R′+R+R′+R	♂+♀+♀+♂	1	R+W′+W+R′	♂+♀+♀+♂	2	R+W′+W+R′	♂+(♂+♀)+♀+♂	1
R′+R+R′+R	♂+♀+♂+♀	1	R+W+R+W	♀+♂+♀+♂	7	**Total**		**108**
						R+W′+R+W′+R′	♀+♂+♀+♂+♀	1

* The color phenotypes are shown from the base to the tip of the blade.

### Sex identification of color sectors and sex boundary in color- sectored F_1_ blades

**Figure 2:**
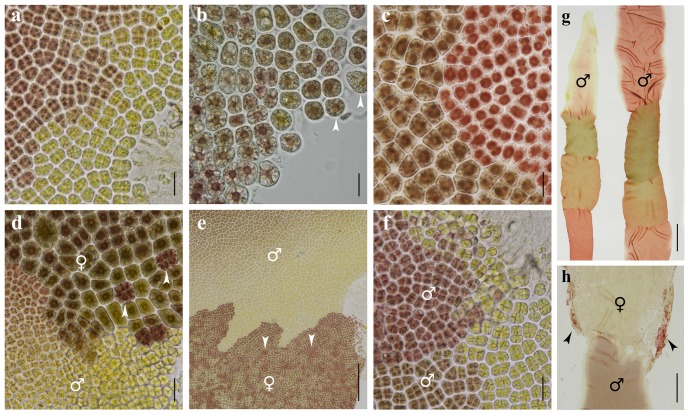
Photomicrographs (*a*–*f*) of male and female sectors near the boundary region between two different color sectors in color-sectored F_1_ blades, and photomacrographs of color-sectored F_1_blades (*g* and *h*), all developed from conchospores of heterozygous conchocelis in the cross between a red mutant (R, ♀) and the wild type (W, ♂) of *Pyropia haitanensis*. *a*. A male near-wild-type sector (W′, ♂) showing spermatangia (yellowish cells). *b*. A female red sector (R, ♀) and released female gametes (arrowheads) when cultured singly. *c*. Vegetative cells in a near-wild-type sector (W′, left) and a red sector (R, right). *d*. A female sector (W, ♀, upper) and a male sector (R, ♂, lower) showing zygotosporangia (arrowheads) and spermatangia (yellowish cells), respectively. *e*. A male sector (R, ♂, upper) and a female sector (W, ♀, lower) showing spermatangia (yellowish cells) and zygotosporangia (red flecks, arrowheads), respectively. *f*. Two male sectors (R, ♂, upper; W, ♂, lower) showing spermatangia (yellowish cells). *g*. Two 4-color-sectored blades showing mature male sectors and immature sectors. *h*. A female sector (W, ♀, upper) and a male sector (R, ♂, lower) showing red zygotosporangia (arrowheads) and spermatangia, respectively. Scale bars: 20 µm in *a*–*d* and *f*, 200 µm in *e*, and 0.5 cm in *g* and *h*.

The results of the single cultures of intact color-sectored F_1_ blades indicated that the maturity of color sectors in a blade was not synchronous (Fig. 2g): In general, male sectors matured earlier than female sectors, and the uppermost sector matured earlier than the middle and basal sectors even though they had the same sex. Male sectors generally matured after 50–70 days of culture, while female sectors generally matured after 65–130 days of culture. Microscopic observation of mature male sectors showed that marginal cells of a sector first became lighter in color, appearing in light yellowish green, original stellate chromatophores disappeared and eventually spermatangia formed after cruciate division (Fig. 2a). The mature female sector was lighter in color, the shape of cells gradually changed from irregular to round or nearly round, and female gametes formed (Fig. 2b). Their original stellate chromatophores became dispersed or small spheroidal (Fig. 2b) and they could be easily distinguished from vegetative cells (Fig. 2c) and spermatangia of a male sector (Fig. 2a). Mature female gametes (Nelson et al. 1999) of female sectors could be fertilized with spermatia released from spermatangia of adjacent male sectors to form zygotosporangia (Fig. 2d, e, arrowheads). Boundaries between adjacent color sectors were zigzag rather than linear, and could be discernible clearly under microscope (Fig. 2d, e). Mature color sectors were unisexual, either male or female, and their boundary always coincided precisely with their sex (Fig. 2d-f). The sex-chimeric pattern of spermatangia and zygotosporangia could be clearly observed between adjacent two color sectors when matured synchronously (Fig. 2d, e, h, arrowheads) in a color-sectored blade.

### Sex analysis of F_1_ gametophytic blades

A total of 584 color sectors excised from 173 color-sectored blades (43 two-sectored, 22 three-sectored, and 108 four-sectored blades) were cultured individually for sex analysis. Most of the color sectors matured after 15–70 days of further culture, but a few did not mature until 90–100 days of culture. Sex observation on these mature color sectors showed that 582 color sectors were all unisexual, male or female, except for 2 color sectors which linearly sectored with male and female parts. In those unisexual sectors, the ratio of female to male was 292∶290, nearly 1∶1. The ratio of parental sex sectors (R♀+W♂) to recombinant sex sectors (R′♂+W′♀) was in accordance with 1∶1 by statistical test (χ^2^ = 0.12, *P*>0.05). However, the sex and color were not genetically linked. According to the sex of each color sector, the sex of 173 color-sectored blades was identified and shown in Table 1. Among them, monoecious blades accounted for 87.9% (152/173) while unisexual blades accounted for 12.1% (21/173, 9 female and 12 male). Monoecious blades accounted for 69.8% of the two-sectored blades, 90.9% of the three-sectored blades, and 94.4% of the four-sectored blades. The percentages of monoecious blades increased significantly with the number of color sectors in color-sectored blades.

Among the 108 four-sectored blades, 100 had two male and two female sectors, and the sex in each blade segregated with a ratio of 1∶1, including 6 sex phenotypes (Table 2). The sex phenotypes of ♂+♂+♀+♀ and ♀+♀+♂+♂ represent the blades produced by first-division segregation (FDS), while the other four sex phenotypes (♂+♀+♀+♂, ♀+♂+♂+♀, ♂+♀+♂+♀ and ♀+♂+♀+♂) represent the blades produced by second-division segregation (SDS). As shown in Table 2, 72% of the blades were produced by SDS; therefore, the sex-determining locus was calculated to be 36 centimorgans (100*72/(28+72)/2) from the chromosomes' centromere. The ratios between two kinds of the sex-sectored blades with mirror symmetric combination were as follows: ♂+♂+♀+♀/♀+♀+♂+♂ = 10/18 (χ^2^ = 1.75, *P*>0.05), ♂+♀+♀+♂/♀+♂+♂+♀ = 17/11 (χ^2^ = 0.89, *P*>0.05), and ♂+♀+♂+♀/♀+♂+♀+♂ = 18/26 (χ^2^ = 1.11, *P*>0.05) (Table 2). Statistical analysis indicated that these ratios were 1∶1, conforming to the characteristics of random separation of chromatids during meiosis.

**Table 2:** Segregation types of sex in the 4-color-sectored F_1_ blades produced in the cross between a red mutant (R, ♀) and the wild type (W, ♂) of *Pyropia haitanensis*.

Sex type	Blade number	Segregation type	
♂+♂+♀+♀	10	First-division segregation (FDS)	Non-crossover (28%)
♀+♀+♂+♂	18	First-division segregation (FDS)	
♂+♀+♀+♂	17	Second-division segregation (SDS)	Crossover (72%)
♀+♂+♂+♀	11	Second-division segregation (SDS)	
♂+♀+♂+♀	18	Second-division segregation (SDS)	
♀+♂+♀+♂	26	Second-division segregation (SDS)	

However, among the remaining 8 blades, 4 had four female sectors (♀+♀+♀+♀), 2 had four male sectors (♂+♂+♂+♂), 1 had one male and three female sectors (♀+♂+♀+♀) and the last one had color phenotype of R+W′+W+R′, although its W′ sector was sectored linearly with male and female sectors and had sex phenotype of R (♂)+W′(♂+♀)+W(♀)+R′(♂) (Table 1). The ratios of female to male in these blades were 4∶0, 0∶4, 3∶1 and 2∶3, respectively. Similar results were obtained also in a 3-sectored blade with color phenotype of R+W+W′, but with sex phenotype of R (♂+♀) +W (♀)+W′ (♂).

### Sex of progeny produced by parthenogenesis of different sectors in color-sectored F_1_ blades

After 20–80 days of further culture, the singly cultured color sectors excised from color-sectored F_1_ blades produced homozygous conchocelis (strain) through parthenogenesis. Careful microscopic examination of over 1000 randomly collected gametophytic blades of each strain found that all blades of each strain were unisexual and unsectored, and had the same color and sex as their original sectors, indicating no sex transfer there.

## Discussion

### Influence of tetrad development on sex phenotype of blades

It was found that the meiosis in *Pyropia* (*Porphyra*) occurs during the conchospore germination, and produces a tetrad which subsequently develops into a genetically chimeric blade [8], [12], [14], [21]. As a result, the survival and development of each cell of the tetrad will deeply influence the segregation of both color and sex of the blade. According to the Mendelian segregation, if the color mutant of *Pyropia* used in the present cross-experiments was caused by nuclear gene mutation(s), regardless it contains one or multiple mutated color genes, parental colors would surely segregate in each F_1_ blade, producing color-sectored blades, and the number of color sectors in a color-sectored blade would be 2 or 4. However, the single-colored blades and color-sectored blades with 3 sectors always appeared in F_1_ blades in the cross experiments between the color mutant and the wild type in *Py. yezoensis* [12], [13], [22], *Py. haitanensis* [8], *P. purpurea* [14], *Py. oligospermatangia* and *Py. katadae* var. *hemiphylla* [21], suggesting that arrested development of the lower cell(s) in the tetrad was probably inherent to blade development and ubiquitous in this genus.

In the present study, observation of early blade development indicated that dominant parts of the blades of *Py. haitanensis* are derived from one or two upper cells of the tetrad, and one or two lower cell(s) of the tetrad stop development after several divisions, forming very small color sectors which contribute to rhizoid formation. In the extreme case, they even become only one cell of the rhizoid without division. Furthermore, microscopic observation of the basal sectors of the color-sectored blades indicates that when the blades were 2–4 mm long, the genotype of R, R′, W and W′ appeared in the basal sector with the same odds (data not shown). However, during the subsequent development of these young blades, the growth of the lowest sector was also arrested. In addition to a relatively low growth rate of the W and W′ sectors, they always become very small sector hardly discerned by naked eyes. While the probability of the lowest sector having genotype of R or R′ which grown into a larger sector in the adult blades due to the remarkable growth advantage of them was much higher than that of W or W′ sectors. Therefore, when the blades reached 3–5 cm long, almost all of the visual basal sectors were R or R′ (Table 1). It suggested that the development of the tetrad cells not only depends on their relative position in a tetrad but also is related to their growth advantage.

The arrested development of the lower cells in the tetrads greatly influences both sex and color phenotypes of the blades. If the development of one lower cell in the tetrads is arrested, the tetrads would develop into three-sex-sectored F_1_ blades (♂+♀+♀, ♀+♂+♂, ♂+♀+♂, ♀+♂+♀, ♀+♀+♂ or ♂+♂+♀), although their sex phenotype remains the same, mainly monoecious one. As a result, the percentage of monoecious blades should not differ significantly between the three- and four-sex-sectored blades, in consistence with our result (*t* = 0.14, *P*>0.01). However, if the development of two lower cells in the tetrads is arrested, the tetrads would lose sex phenotypes of two sectors and develop into dioecious (unisexual) blades (♂+♂ or ♀+♀) or monoecious blades (♂+♀ or ♀+♂). This probably is the main reason why the proportion of unisexual blades reached 30.2% among the two-sex-sectored blades (Table 1). If the unisexual two-sex-sectored blades (♂+♂ and ♀+♀) are really caused by the arrested development of two lower cells in the tetrads, the percentage of monoecious blades would increase from 87.9% to 95.4% (165/173) in Table 1. In the present study, the percentage of 4-sectored blades was very low, but they represented the perfectly developed meiotic tetrad. Therefore, sex analysis in the present study was mainly performed in 4-sectored blades.

### Observation results on wild *Py. haitanensis* blades probably reflect only the sex of a part of the blades

The previous study found that 99.9% of wild blades of this species were dioecious and only 0.1% was monoecious when examined by naked eyes [7], which is inconsistent with the results of the present cross experiment and can probably be explained as following: (1) The wild blades of *Py. haitanensis* were produced by sexual reproduction, its meiosis occurred during conchospore germination, and the produced tetrads inevitably developed into chimeric blades composed of linearly arranged sex sectors. However, the sex-sectored blades could not be identified owing to the lack of distinct markers in the wild blades. In addition, it was erroneously considered that in *Py. haitanensis* the meiosis occurs at/before the formation of conchospores and the blade is mainly dioecious [3]; as a result, the sex of wild blades was often judged only by spermatangia or zygotosporangia appeared in the tip part of blades, without realizing that the wild blades are sex-sectored and the different sex sectors mature asynchronously in a blade. Thus, it is considered that previous results probably reflect only the sex phenotype of some but not all of sex sectors in the blades. (2) For wild blades of *Py. haitanensis*, once the spermatangia or zygotosporangia either at the tip or in the middle part of blades matured, they are always washed away or mutilated by the seawater. Thus, it is usually impossible to get intact wild blades, and the blade sex is usually to be determined only by the remaining spermatangia or zygotosporangia. Meanwhile, the tip, middle and basal parts of a blade mature asynchronously, and this could be another reason for the difficulty to obtain monoecious blades. Moreover, almost all of the wild blades collected from rocks are non-intact, because the rhizoids of blades strongly stick to uneven rock surface and are difficult to obtain blades including intact basal parts. Therefore, to know the real sex of wild blades, it is necessary to obtain intact blades and let them mature completely. Unfortunately, nearly 100% of the mature wild blades sampled from the nature in the past several years were incomplete. (3) The present cross experiment indicated that the arrested development of lower cell(s) in the tetrad of *Py. haitanensis* would decrease the number of observable sex sectors and lead to misjudge their sex, and the upper cells of tetrads would divide to form the dominant part of blades in *Py. haitanensis* [8]. Together, these reasons could partially explain why monoecious blades in wild populations of *Py. haitanensis* are not easy to be observed.

### Sex determination mechanism

Mitman and van der Meer [14] indicated that meiotic segregation of a pair of alleles in *P. purpurea* is the controlling factor of its sex. In the present study on *Py. haitanensis*, it was shown that (1) the segregation ratio of female and male was 1∶1; (2) almost every color sector of color-sectored F_1_ blades was unisexual; and (3) the boundaries for sex and color always coincided precisely. These results are in agreement with those obtained in the cross experiment of *P. purpurea* by Mitman and van der Meer [14], suggesting that the sex of *Py. haitanensis* is also controlled by meiotic segregation of a pair of alleles.

### Analysis of aberrant segregation of parental sex and color

Aberrant segregations at ratios of 0∶4, 4∶0, 3∶1 and 2∶3 of the female and male in a few of four-sectored blades were observed in the present study. Because each blade of *Pyropia* (*Porphyra*) was chimeric and composed of four meiotic products, theoretically, it should have no more than four sectors [8], [13]–[15], [21], [23]. However, blades containing five color sectors, which were linearly sectored with five sex sectors, were observed in the present study. Moreover, aberrant segregations of parental colors were also observed in a few of four-sectored blades, such as R′+R+R′+R (4∶0) and R′+R+W+R (3∶1). Similar aberrant segregations have also been found in tetrad analysis of Ascomycetes fungi [24]–[26] and caused by gene conversion [24], [26]–[28]. Gene conversion in fungi could produce a meiotic tetrad that segregates at a 1∶3/3∶1 ratio. The rarer ratios of 4∶0/0∶4 are presumably resulted from double or multiple gene conversion events [29], [30]. Half-chromatid conversion (or post-meiotic segregation) would have a 3∶5/5∶3 ratio [31], [32].

**Figure 3:**
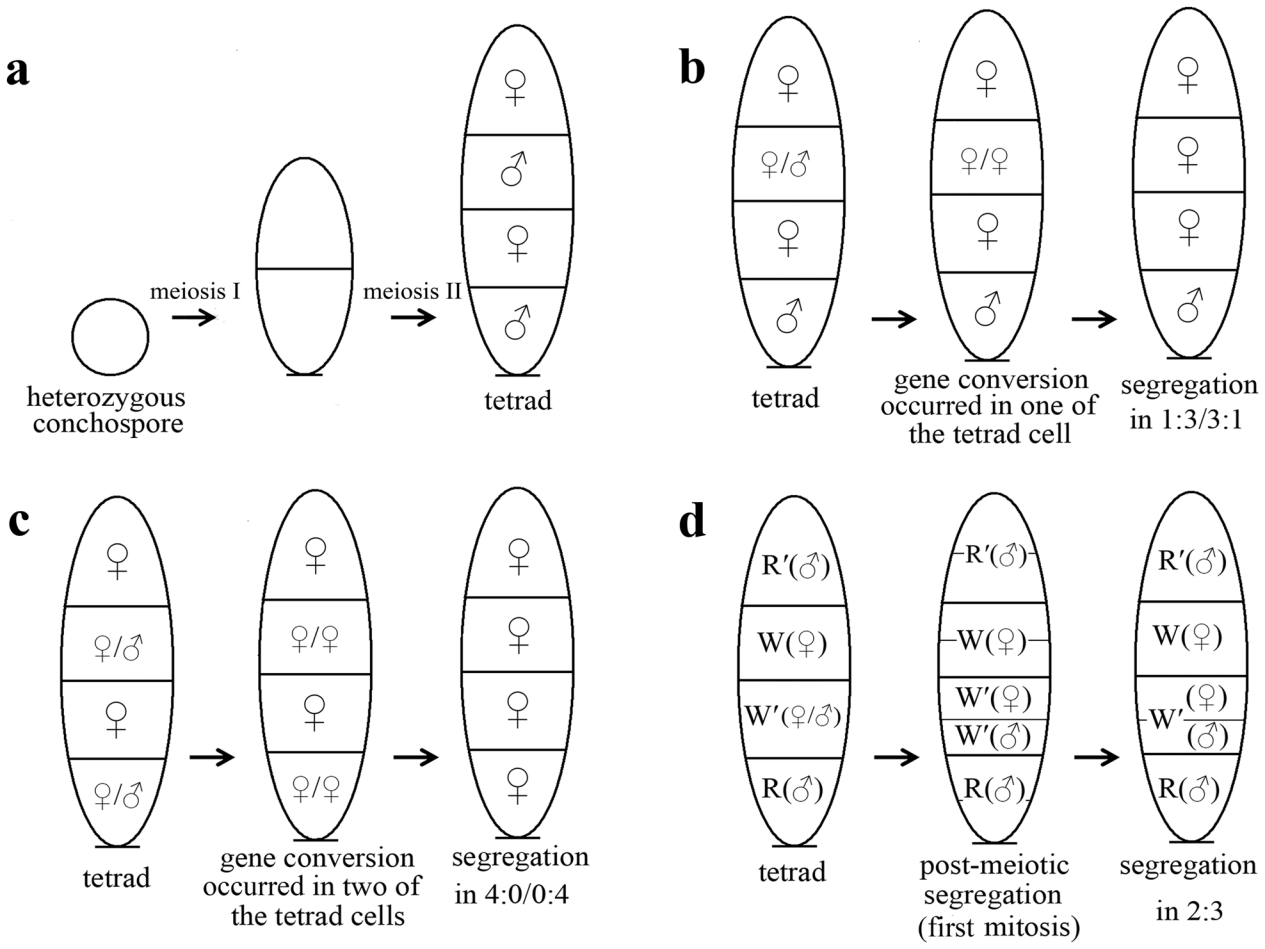
Diagrammatic illustration of the conversion of sex-determining gene in *Pyropia haitanensis*, leading to aberrant segregation of sex. *a*. As the sex locus of *Py. haitanensis* is distant from the centromere (36 cM), most crossover events occur between the sex locus and the centromere, and the parental sex in F_1_ blades segregates normally in a ratio of 1∶1. *b*. When crossover occasionally occurs in the sex-determining gene or nearby locus, heteroduplex mismatch (shown as ♀/♂) produced by recombination is repaired by male or female parent side of single-stranded DNA, which is used as a template (shown as ♀/♀), and a single gene conversion event would result in an aberrant sexual segregation in a ratio of 3∶1 or 1∶3. *c*. When crossover occasionally occurs in the sex-determining gene or nearby locus, two individual gene conversion events would result in an aberrant sexual segregation in a ratio of 4∶0 or 0∶4. *d*. A half-chromatid conversion event would result in post-meiotic segregation of the sex determining gene, producing a color sector with two sexes, such as R(♂)+W′(♂+♀)+W(♀)+R′(♂).

The red color mutant used in the present study was caused by nuclear gene mutations [8]. Like the wild type, its sex is also controlled by nuclear gene. Thus, the aberrant segregation of color and sex appeared in the present experiment is not due to maternal inheritance and organelle inheritance. Because the meiotic tetrads of *Py. haitanensis* and fungi showed very similar characteristics in tetrad patterns, we analyzed them using the same method. As a result, we speculate that similar gene conversion events probably occur in *Py. haitanensis* during the meiosis, resulting in aberrant egregation of sex alleles in F_1_ blades (Fig. 3). The aberrant segregations of parental colors and sex probably have the same mechanisms.

Furthermore, studies on an artificial recombination-initiating region (*MAT* locus) with *HO*-induced cutting in yeast showed that among 1550 tetrads, 215(14%) had a ratio of 4α∶0a; 9 had 1α∶3a and 19 had 3α∶1a when the *HO* gene was expressed, while only a few aberrant segregations with a ratio of 1α∶3a at *MAT* locus when the *HO* gene was not expressed [33]. Similar results were also found at *HIS4* locus [33]. It suggested that the different sensitivity of two alleles in a heterozygote to an endonuclease can cause extreme disparity of gene conversion. Moreover, in the budding yeast *Saccharomyces cerevisiae*, mother cells but not daughter cells can switch mating type by *HO*-induced mitotic gene conversion [34]. Therefore, whether the aberrant segregations of the sex and/or color in the present study have similar mechanism to them still has to make further study, and more studies are also need to investigate whether aberrant cell divisions occur during formation of the tetrad of the germinating conchospores.

## Conclusions

(1) The sex of *Py. haitanensis* is controlled by a pair of sex alleles. Although the sex destiny of the tetrad produced by meiosis of conchospore has been determined, the full expression frequency of sex phenotype for all cells in the tetrad is low because one or two lower cell(s) of the tetrad mainly contribute to rhizoid formation. (2) Most gametophytic blades of *Py. haitanensis* produced by sexual reproduction are monoecious, and only a few are dioecious. (3) Gene conversions rather than sex transfer probably occur in *Py. haitanensis*.
